# miR-100-5p activation of the autophagy response through inhibiting the mTOR pathway and suppression of cerebral infarction progression in mice

**DOI:** 10.18632/aging.204971

**Published:** 2023-08-22

**Authors:** Xiaoyun Cao, Xiangjian Zhang, Junmin Chen, Qian Sun, Yufan Sun, Na Lin, Xiaoxia Liu

**Affiliations:** 1 Neurology Department, The Third Hospital of Hebei Medical University, Shijiazhuang, China; 2 Hebei Collaborative Innovation Center for Cardio- Cerebrovascular Disease, Shijiazhuang, China; 3 Hebei Key Laboratory of Vascular Homeostasis, Shijiazhuang, China; 4 Neurology Department, The Second Hospital of Hebei Medical University, Shijiazhuang, China; 5 Institute of Clinical Medicine, Hebei Medical University, Shijiazhuang, China; 6 Neurology Department, Shijiazhuang Hua Yao Hospital, Shijiazhuang, China

**Keywords:** miR-100-5p, cerebral infarction (CI), mTOR, autophagy, apoptosis

## Abstract

In recent years, the association between microRNAs (miRNAs) and autophagy in cerebral infarction (CI) has attracted increasingly more attention. The mammalian target of the rapamycin (mTOR) pathway is a key protein regulating the autophagy response. miR-100-5p can bind to the mTOR protein, but its role in CI remains unclear yet. This experiment aims to clarify the role of miR-100-5p in CI. Bioinformatics analysis was performed to screen differentiated expressed functional genes between CI tissue and normal tissue specimens. *In vivo* experiments: the mouse model of CI was established by middle cerebral artery occlusion (MCAO) methods, After being treated with miR-100-5p-overexpressing lentivirus, the amount of terminal deoxynucleotidyl transferase (TdT) dUTP nick-end labeling (TUNEL)-positive fluorescence and the fluorescent expression level of mTOR protein were significantly inhibited in the CI region. Western blotting analysis showed that miR-100-5p inhibited the protein expression level of phosphorylated mTOR and total mTOR and enhanced the expression of autophagy-related proteins Beclin, microtubule-associated protein light chain 3II (LC-3II), and autophagy-related gene 7 (ATG-7). For *in vitro* experiment, after the BV-2 cells were successfully infected with the control lentivirus and miR-100-5p-overexpression lentivirus, they were stimulated with 1% hypoxia and low-glucose medium in a tri-gas incubator for 24 h. It was found that miR-100-5p could significantly lower the protein expression level of phosphorylated mTOR and total mTOR, and increase the expression of the Beclin, LC-3II, ATG-7 autophagy related proteins. miR-100-5p promotes the autophagy response through binding to mTOR protein, thereby inhibiting apoptosis and delaying the progression of CI.

## INTRODUCTION

Cerebral infarction (CI) is defined as localized ischemic necrosis or the softening of brain tissues due to impairment of the blood supply to the brain, hypoxia, or ischemia [1, 2]. CI can be clinically classified into cerebral thrombosis, lacunar infarction, and cerebral embolism, accounting for 80% of all stroke cases [3]. In older adults, CI contributes 50% to 80% of all cerebrovascular diseases [4]. The regulatory roles of miRNA in various pathways, including cell proliferation and apoptosis, fat metabolism, hematopoietic process, and development, have been elucidated. Since the discovery of microRNAs (miRNAs), their important role in each link of the pathogenesis of atherosclerosis has been clarified in numerous studies [2, 5]. Specific miRNAs may be key regulatory genes in the vascular pathophysiological process, including vascular cell proliferation, autophagy, oxidative stress, and inflammatory response [6, 7]. Research has reported that miRNA plays a regulatory role in cerebrovascular infarction [8]. It has been found that miR-210 can facilitate proliferation and inhibit the apoptosis of vascular smooth muscle cells, thereby promoting cell growth [9]. The abnormal miR-100-5p has been reported to participate in cerebral disease including glioblastoma [10], neurodegeneration [11], and tumors in the central nervous system [12]. Moreover, decreased miR-100 was reported to regulate ischemia in stroke and blood vessels [13, 14]. The above evidence collectively reveals that miR-100 plays a regulatory role in cerebral ischemic disease. Differentially expressed genes (DEGs) in CI have been identified in bioinformatics studies, which reported that miR-100-5p had a low expression in diseases and was enriched in pathways such as the mammalian target of rapamycin (mTOR). mTOR is a regulatory molecule involved in a variety of signaling pathways, which is an important player in cell apoptosis and autophagy [15, 16]. The mouse cerebral ischemia model is widely used to simulate human cerebral ischemia [17, 18]. In the present study, C57BL/6J mice were used as the subjects, and the role of miR-100-5p in the pathological model of CI mice and its related regulatory mechanism were explored. We present the following article in accordance with the ARRIVE reporting checklist (available at https://atm.amegroups.com/article/view/10.21037/atm-23-463/rc).

## RESULTS

### Bioinformatics analysis

After a searching and standardized screening of the CI-related data sets, the GSE102541 and GSE86291 expression profile microarrays were determined as the study samples. GSE102541 and GSE86291 were downloaded from the GEO official website using Bioconductor in R software.

### Screening results of DEGs

**Figure 1:**
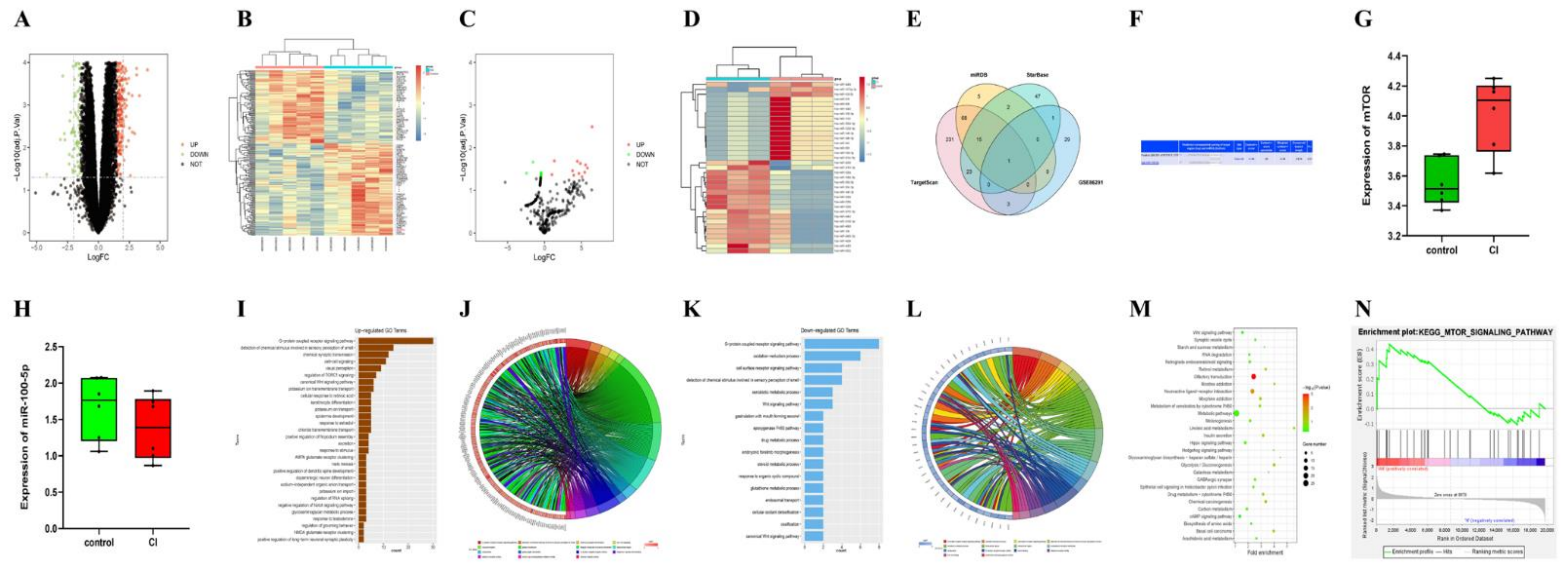
**The bioinformatics analysis results of GSE102541 and GSE86291.** (**A**, **B**) The DEGs of GSE102541 are presented in the volcano plots and cluster analysis diagrams. (**C**, **D**) The volcano plots of GSE86291 (the downregulated DEGs included miR-100) and cluster analysis diagrams. The Venn diagram (**E**) predicted using miRDB, starBase, and TargetScan, shows the intersection of miRNAs targeted mTOR, and the shared miRNA (miR-100-5p) with GSE86291. (**F**) The binding sites between miR-100-5p and mTOR. (**G**, **H**) The expression of mTOR and miR-100-5p in CI and the control tissue of GSE102541. (**I**, **J**) The GO enrichment analysis charts and GO chord graphs of GSE102541. (**K**, **L**) The GO enrichment analysis charts and GO chord graphs of downregulated genes in GSE102541. (**M**, **N**) The KEGG enrichment analysis charts and GSEA chart of GSE102541. DEG, differentially expressed gene; miRNA, microRNA; CI, cerebral infarction; GO, Gene Ontology; KEGG, Kyoto Encyclopedia of Genes and Genomes; GSEA, gene set enrichment analysis; mTOR, mammalian target of the rapamycin.

After the quantile normalization of GSE102541 and GSE86291 with the “limma” package in R software, the analysis of DEGs (|log2 foldchange FC|<1, *P*<0.05) was completed, relevant DEGs were obtained (the upregulated DEGs included mTOR in the CI group; the downregulated DEGs included miR-100-5p in the CI group, Figure 1A–1D). The volcano plots and cluster analysis diagrams of GSE102541 (Figure 1A, 1B) and GSE86291 (Figure 1C, 1D) were drawn. The association between the miRNAs and mTOR was predicted using miRDB, starBase, and TargetScan, and a Venn diagram was plotted (Figure 1E) to find shared miRNA with GSE86291. It was found that miR-100-5p was included in the intersection, indicating that miR-100-5p has an association with mTOR. Their binding sites are shown in Figure 1F. The expressions of mTOR and miR-100-5p in GSE102541 are listed in Figure 1G, 1H.

### Metabolic pathway and biological process analysis of key targets

GO enrichment analysis was conducted on the upregulated genes in GSE102541 using DAVID, and the GO enrichment analysis charts (Figure 1I) and GO chord graphs (including the TORC1, a ternary complex composed by mTOR, RAPTOR (mTOR regulation related protein) and G-Beta L (G-protein β subunit-like protein); Figure 1J) were plotted using R language. GO enrichment analysis was conducted on the downregulated genes in GSE102541 using DAVID, and the GO enrichment analysis charts (Figure 1K) and GO chord graphs (Figure 1L) were plotted using R language. Additionally, KEGG enrichment analysis was conducted on GSE102541, and the KEGG enrichment analysis charts (Figure 1M) were plotted, confirming the enrichment in the relevant pathway. After gene set enrichment analysis (GSEA) and GSEA chart plotting (Figure 1N), the enrichment in relevant pathway was also confirmed.

### Overexpression of miR-100-5p could inhibit CI

**Figure 2:**
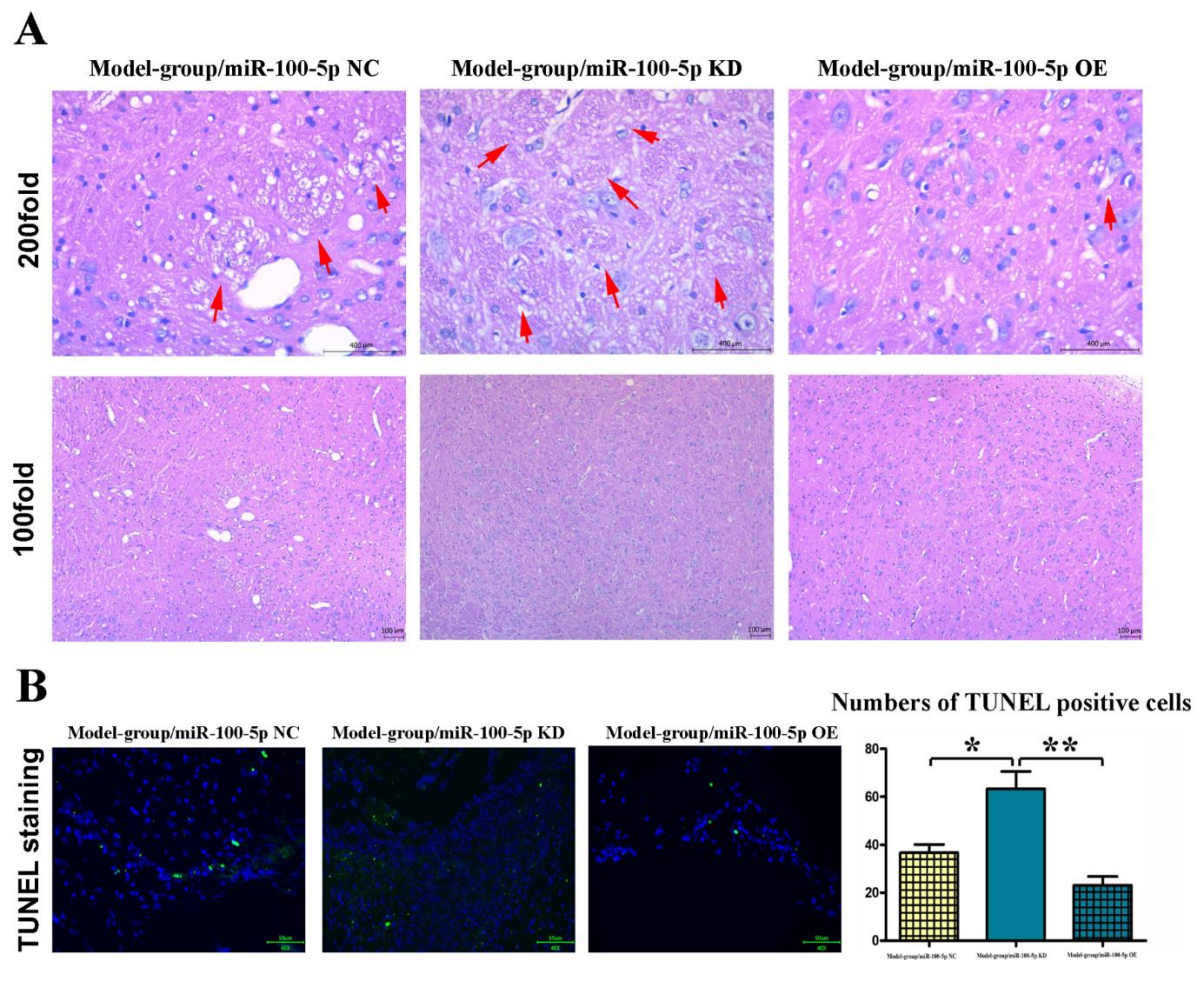
**Overexpression of miR-100-5p could inhibit CI in mice.** (**A**) HE staining results of the model, miR-100-5p KD, and miR-100-5p OE groups. In CI model group, a large number of ethmoid necrotic foci in ischemic cerebral tissue could be observed (arrowheads). The number of neurons in the necrotic foci was significantly reduced, with pyknosis and hyperchromatic nuclei; miR-100-5p KD aggravates the damage in cerebral while miR-100-5p OE attenuates the damage caused by CI. The CI volume was significantly reduced in the miR-100-5p OE group compared with the model group. (**B**) Overexpression of miR-100-5p inhibited apoptosis in the brain tissue of mice. TUNEL staining was performed, and the number of TUNEL-positive cells significantly declined in the miR-100-5p OE group compared with the model group. CI, cerebral infarction; HE, hematoxylin and eosin; NC, negative control; KD, knock down; OE, overexpression; TUNEL, terminal deoxynucleotidyl transferase (TdT) dUTP nick-end labeling. *P<0.05, **P<0.01.

Hematoxylin and eosin (HE) staining was used to assess CI. Varying degrees of CI occurred in the model group (Figure 2A). Compared with that in model group, the CI volume was significantly reduced in the miR-100-5p–OE group (*P*<0.01).

### Overexpression of miR-100-5p could reduce the TUNEL-positive fluorescence and inhibit apoptosis

The results of TUNEL staining showed that CI could cause the apoptosis of brain tissues in mice (Figure 2B). Compared with that in model group, the number of TUNEL-positive cells significantly declined in the miR-100-5p–OE group (*P*<0.01; Figure 2B), indicating that overexpression of miR-100-5p can significantly reduce CI-induced apoptosis in mice.

### Overexpression of miR-100-5p could suppress the protein expressions of phosphorylated mTOR and total mTOR while enhancing the expressions of autophagy-related proteins Beclin, microtubule-associated protein light chain 3II (LC-3II), and ATG-7

**Figure 3:**
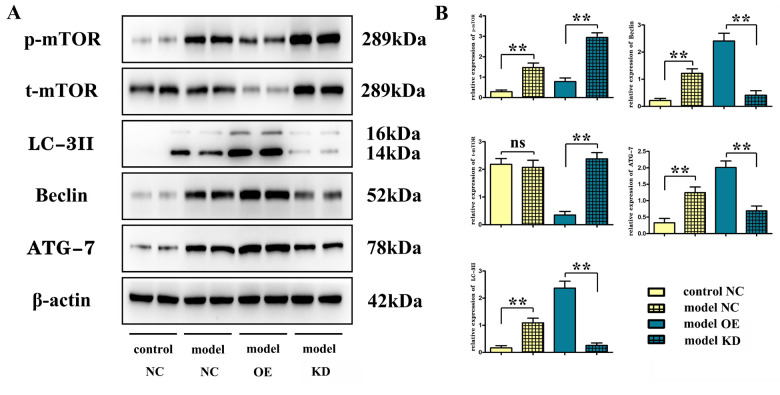
**Overexpression of miR-100-5p decreased the protein expressions of phosphorylated mTOR and total mTOR but increasing the expression of autophagy-related proteins Beclin, LC-3II, and ATG-7 *in vivo*.** (**A**) Representative Western blot images. (**B**) Column comparison of the relative expression of proteins. mTOR, mammalian target of the rapamycin; LC-3II, microtubule-associated protein light chain 3II; ATG-7, autophagy-related gene 7; NC, negative control; KD, knock down; OE, overexpression. *P<0.05, **P<0.01. NS: irrelevant.

**Figure 4:**
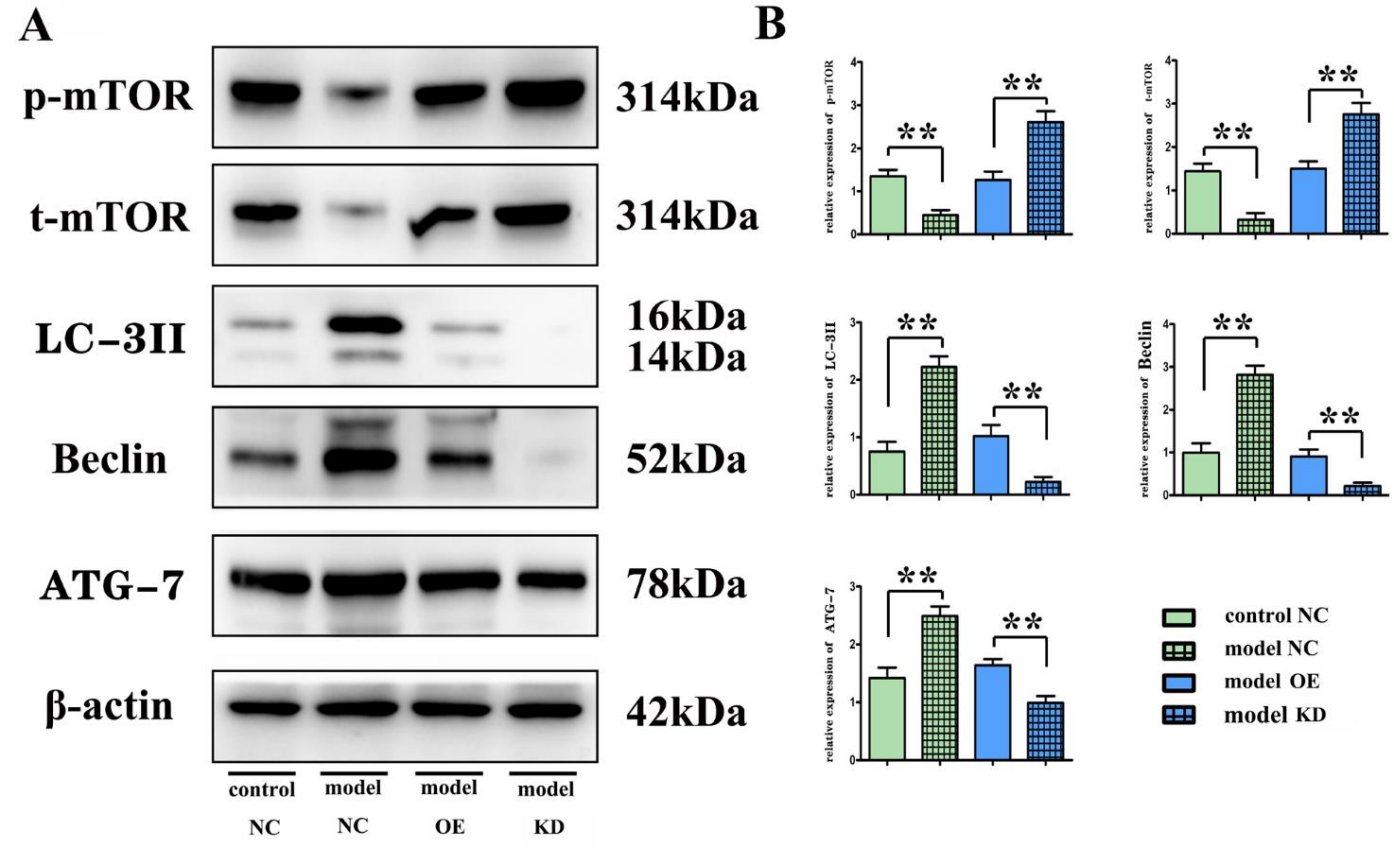
**The *in vitro* BV-2 cell experiment. Overexpression of miR-100-5p (OE) decreased the protein expressions of phosphorylated mTOR and total mTOR, while increasing the expressions of autophagy-related proteins Beclin.** (**A**) Representative Western blot images. (**B**) Column comparison of the relative expression of proteins. LC-3II, and ATG-7 compared with the empty vector group (NC) and miR-100-5p inhibitor group (KD). mTOR, mammalian target of the rapamycin; LC-3II, microtubule-associated protein light chain 3II; ATG-7, autophagy-related gene 7; NC, negative control; KD, knock down; OE, overexpression. **P<0.01.

Western blotting indicated that the miR-100-5p–OE group had significantly decreased protein expressions (Figure 3A, 3B) of phosphorylated mTOR and total mTOR (*P*<0.01), along with significantly enhanced expressions of autophagy-related proteins Beclin, microtubule-associated protein light chain 3II (LC-3II), and ATG-7, compared with the model group (*P*<0.01). For the *in vitro* BV-2 cell experiment, the protein expressions (Figure 4A, 4B) of phosphorylated mTOR and total mTOR significantly decreased (*P*<0.01) while the expressions of autophagy-related proteins Beclin, LC-3II, and ATG-7 significantly enhanced in the miR-100-5p–OE group compared with the empty vector group (*P*<0.01).

## DISCUSSION

CI, one of the common frequently occurring diseases in the nervous system, is associated with high mortality and disability rates [19] and seriously threatens the life safety of patients. Currently, cerebral ischemia/reperfusion injury is the major cause of acute CI [2, 20, 21]. The pathogenesis of CI is complex, and it has been shown that it is related to excessive production of free radicals, the toxic effect of excitatory amino acids, intracellular calcium overload, inflammatory response, and autophagy [21–23]. miRNAs play important roles in the regulation of CI, and their expression levels are closely related to the damage and repair of CI [24, 25]. In the present study, the CI-related data sets were searched through bioinformatics, downloaded from the GEO official website of the National Center for Biotechnology Information (NCBI), and the DEGs were screened. miR-100-5p has been reported to be involved in the apoptotic pathway in chronic traumatic encephalopathy [26]. It was found that miR-100-5p had a low expression but that mTOR had a high expression in CI. As a conserved serine/threonine protein kinase [27], mTOR serves as an important protein in regulating cell growth, proliferation, movement, survival, and autophagy [16, 28].

Furthermore, the association between miRNAs and mTOR was confirmed by miRDB, starBase, and TargetScan. Other research has found that miR-100-5p can be involved in autophagy through targeting mTOR in stroke, prostate cancer, and neurodegeneration [11, 13, 29]. However, the regulatory mechanism of miR-100-5p in CI has been rarely reported on. In this study, through *in vivo* and *in vitro* experiments and an MCAO mouse model, the regulatory effect of miR-100-5p in CI and its mechanism were examined. The results showed that severe CI was accompanied by apoptosis in the model group. After overexpression of miR-100-5p, the CI area was significantly reduced in the model group. The results of TUNEL staining revealed that overexpression of miR-100-5p could significantly suppress CI-induced neuronal apoptosis and promote autophagy.

Recently, postcerebral injury autophagy has become a research hotspot. The molecular regulatory mechanism of autophagy is complex, involving a variety of molecules and genes, such as ATG and LC3. Beclin-1 is another autophagy-related protein and was the first major protein reported to be implicated in the initiation of autophagy [30]. In this study, overexpression of miR-100-5p significantly reduced the protein expression levels of phosphorylated mTOR and total mTOR but increased the expression levels of autophagy-related proteins Beclin-1, LC-3II and ATG-7. It can be seen that overexpression of miR-100-5p can promote the autophagy response through binding to mTOR protein, thereby inhibiting apoptosis and delaying the progression of CI. To further verify the mechanism of action of miR-100-5p in CI, BV-2 cells were transfected with miR-100-5p–OE lentivirus. The results of TUNEL staining suggested that miR-100-5p could increase the number of autophagosomes, thus suppressing apoptosis. Moreover, according to the results of Western blotting, miR-100-5p could negatively regulate the transcription and phosphorylation level of mTOR protein and upregulate the expression levels of autophagy-related proteins.

The limitations of our study cannot definitively indicate that miR-100-5p regulates autophagy through inhibition of the mTOR pathway, and the conclusions are preliminary. Strictly designed *in vivo* studies are needed to clarify the effect of miR-100-5p on CI, including the degree to which it can improve cerebral blood flow and brain function.

## CONCLUSIONS

In conclusion, miR-100-5p was found to alleviate the nerve injury in CI via a mechanism which may involve the promotion of autophagy response through binding to mTOR protein, thereby inhibiting apoptosis.

## MATERIALS AND METHODS

### Bioinformatics analysis

The microarrays were screened with the Gene Expression Omnibus (GEO) database (http://www.ncbi.nlm.nih.gov/geo/). The microarrays enrolled were required to contain CI tissue specimens and normal tissue specimens and to share the same platform. The study was conducted in accordance with the Declaration of Helsinki (as revised in 2013).

### Screening of DEGs

After the target microarray was determined, differential analysis was conducted on the standardized microarray expression profile using the “limma” package in R software (The R Foundation for Statistical Computing), and multiple tests and corrections were performed using the Bayesian method, which was followed by the screening of DEGs [|log2foldchange (FC) |>1 and *P*<0.05]. The screening results were visualized through the “pheatmap” and “ggplot2” packages in R, and output in the form of volcano plots and heat maps.

### Gene function annotation and pathway enrichment analysis

The DEGs obtained were imported into the Database for Annotation, Visualization and Integrated Discovery (DAVID) 6.8 database (https://david.ncifcrf.gov/), and Gene Ontology (GO) enrichment analysis and Kyoto Encyclopedia of Genes and Genomes (KEGG) enrichment analysis were carried out on the upregulated and downregulated DEGs in GSE102541. The results were output in the form of enrichment analysis charts and chord graphs. All publicly available database information was obtained in September 2022.

### Main reagents and machines

The following reagents and devices were used: terminal deoxynucleotidyl transferase (TdT) dUTP nick-end labeling (TUNEL) cell apoptosis assay kits (Beyotime Biotechnology Co., Ltd., Shanghai, China), Western blotting kits (Abcam, Cambridge, UK), miR-100-5p–overexpression lentivirus (RiboBio Co., Ltd., Guangzhou, China), a stereotaxic apparatus, mouse middle cerebral artery occlusion (MCAO) thread, animal anesthesia machine and isoflurane (RWD Biotechnology Co., Ltd., Shenzhen, China), an electronic balance (Beijing Sartorius Instrument and System Engineering Co., Ltd., Göttingen, Germany), a 5804R tabletop high-speed refrigerated centrifuge (Eppendorf, Hamburg, Germany), and a gel imaging system (Syngene, Bangalore, India).

### Grouping and MCAO modeling

A total of 20 specific pathogen-free (SPF) male C57BL/6J mice weighing 20–25 g at 8 to 10 weeks-of-age were purchased from the Laboratory Animal Center of Hebei Medical University. The mice were divided into a control, model group, miR-100-5p inhibitor (KD), and miR-100-5p–overexpression (OE) lentivirus group, with 5 mice in each group. The mice were kept in a sterile room, given adequate food and water, and then used in follow-up experiments after a week of adaptive feeding. They were deprived of food but not water for 12 h before operation in a sterile environment. After anesthesia with isoflurane, the mice were fixed on the operating table in a supine position, and the neck skin was prepared and disinfected with iodophor. A median neck incision was made, from which the subcutaneous fat and muscle, the right common carotid artery (CCA), external carotid artery (ECA), and internal carotid artery (ICA) were carefully separated. The CCA and ICA were temporarily clamped using a micro-arterial clamp, and the thread was prepared without tightening at the distal end of the CCA. The main ECA was separated, and the thyroid artery and occipital artery were bluntly separated and cut off by fulguration; the ECA was then pulled down to align it in a straight line with the ICA. A small oblique incision was made with ophthalmic scissors about 2 mm away from the distal end of the CCA bifurcation, from which the heparin-soaked thread (0.26 mm in diameter) was inserted, and the suture line was tightened. The ICA clamp was loosened, and the thread was slowly pushed from the ICA into the cranial artery branch. MCAO was deemed successful when the sense of resistance was felt at about 2.0 cm away from the bifurcation of the ICA and ECA. The prepared thread was then tightened, and the start time of embolism was recorded. A small amount of penicillin injection powder was applied to the surgical wound, the subcutaneous soft tissues and skin were aligned and sutured, and the tail of the thread was left outside the body. After 2 h, the thread was slowly withdrawn to the CCA incision to restore the blood supply to the MCAO region. During operation, the anal temperature of mice was maintained at about 37° C until resuscitation. Lentivirus vector harboring miR-100-5p sequences (109 TU/mL) was mixed with the cationic lipid polybrene and injected to intracerebroventricular 10 mins after MCAO. A protocol was prepared before the study without registration.

### Observation of the pathological changes in the brain tissue of each group of mice

After decapitation of the mice, the brain tissue was fixed with 4% paraformaldehyde, conventionally dehydrated, soaked in wax, embedded in paraffin, sliced at 4-μm, and then displayed and baked. The tissue sections were placed in xylene (I, II) for 10 min, dewaxed for 5 min, rinsed with tap water, stained with hematoxylin for 4 min, washed with distilled water for 3 min. The section was differentiated with 1% hydrochloric acid, and washed with water and then immersed by 1% ammonia inverse blue for "blueness" of color. The sections were washed again with water and then stained by 0.5% eosin solution for 1 min, rinsed with water again, and dehydrated quickly. The film was mounted with neutral gum and observed, with pictures being taken under an optical microscope.

### Detection of neuronal apoptosis with TUNEL assay

At 24 h after operation, the brain was harvested and prepared into brain tissue sections. The sections were baked at 60° C for 1 h, deparaffinized with xylene, dehydrated with gradient alcohol, treated with proteinase K at room temperature for 20 min, mixed with TUNEL working solution (containing TdT buffer with TdT, fluorescent labeling solution, and TUNEL test solution) following manufacture’s instruction (C1088, One-step TUNEL apoptosis detection kit, Beyotime, Shanghai, China), and incubated at 37° C for 1 h. The sections were then washed with phosphate-buffered saline (PBS) and sealed with antifade mounting medium. Then the TUNEL staining results were observed and photographed under a fluorescence microscope, and the neuronal apoptosis rate was calculated as follows: number of apoptotic cells/total number of cells ×100%.

### Detection of mTOR, Beclin, microtubule-associated protein light chain 3II, and ATG-7 protein expressions with Western blotting

At 24 h after operation, the brain was harvested and placed in liquid nitrogen for Western blotting. The cortex tissues on the ischemic side were harvested and homogenized, which was followed by centrifugation at 12,000 rpm at 4° C for 20 min. The protein was then extracted from the supernatant, and its concentration was measured with bicinchoninic acid assay (BCA). An appropriate amount of protein loading buffer was mixed, centrifuged, denatured and loaded. After separation with sodium dodecyl-sulfate polyacrylamide gel electrophoresis (SDS-PAGE) (50 μg/well), the protein was transferred onto a membrane, sealed, and incubated with primary antibodies at 4° C overnight and with secondary antibodies at room temperature for 2 h on a shaking table. The images were then acquired using a Bio-Rad imager (Bio-Rad Laboratories, Hercules, CA, USA), and the optical density was measured using Image Lab Software (Bio-Rad Laboratories). The relative protein expression was expressed as the ratio of optical density of the target protein to that of β-actin.

### Cell transfection

The BV-2 cells were inoculated into a 6-well plate (1×10^5^/well). When 80% of the plate was covered with cells, the medium was replaced with serum-free medium for culture for another 24 h. Following this, lentivirus vector harboring negative control sequence (miR-100-5p NC) and miR-100-5p mimic sequence (miR-100-5p OE, about 1.2 to 1.6-fold upregulated, compared with NC group) was transfected to cells. The transfected concentration of miR-100-5p was 30 pmol. In addition, lentivirus harboring an inhibitor of miR-100-5p (miR-100-5p KD) were prepared into a complex and transfected into cells (1×10^5^/mL) according to the instructions of ViraDuctin™ (Cell Biolabs, San Diego, CA, USA). The medium was then replaced, and the cells were cultured for 48 h. The cells were divided into an empty vector group and an miR-100-5p–OE group for later experiments.

### Statistical analysis

SPSS 20.0 software (IBM Corp., Armonk, NY, USA) was used for data analysis. All data are expressed as mean ± standard deviation $\left(\bar{\text{x}}\pm \text{s}\right)$ Analysis of variance (ANOVA) was used for statistical analysis of the difference between multiple groups, and T-test was used for statistical analysis of the difference between two groups. *P*<0.05 was considered to be statistically significant. The parallel experiment was repeated for more than three times in each group.
